# New Methods of Treatment in Phthisis

**Published:** 1891-11-14

**Authors:** 


### The Practitioner's Mirror and Retrospect.

*[The Editor will be glad to receive offers of co-operation and contributions from members of the profession. All letters should be addressed to* The Editor, The Lodge, Porchester Square, London, W.]

### MEDICINE.

### NEW METHODS OF TREATMENT IN PHTHISIS.

VII.—The Lannelongue Treatment of Tubercular Joints and Bones.

On the 6th of July Dr. Lannelongue made a communication to the Paris Academy of Medicine on a new method of treating tuberculosis. The essential feature of this new method consists in the injection into the tissues in the neighbourhood of and around the disease a solution of chloride of zinc, the effect of which, the originator claims, is to produce a transformation of morbid tissue, and the almost immediate formation of connective tissue. His attention was, in the first place, directed to this idea by observing the remarkable effects obtained by injections of chloride of zinc in a case of congenital hypertrophy of the arm in a child a few months old. In the course of a few months the flabby moist tissues of this overgrowth were transformed by the treatment into a dense hard tissue. Moreover, these effects were produced, not only at the seat of injection, but radiated as from so many centres. Dr. Lannelongue divided his communication under three headings—the clinical, experimental, and technical. The object in view is to sclerose the tuberculous tissue wherever it may be located, and in this manner to endeavour to bring about the condition of things least favourable to the further development of the micro-organism on the assumption that this morbid agent disappears, or becomes impotent for evil when this condition is realised. The method then consists in causing the therapeutic agent to penetrate, not into the granulations nor the foci themselves, but only outside and around them.

In speaking of the technique of this sclerogenous method, M. Lannelongue emphasises the principle on which it is based, viz., to act upon the zone of tissues in the immediate neighbourhood of the tubercular masses and new growths, that is to say upon the parts which contain the vessels nourishing the tubercular tissue. Leaving out of consideration the exceptional cases, he ordinarily uses a solution of 1 in 10, and he injects two or three drops in a selected spot; the operation is repeated several times. In this way he makes six, eight, or ten injections at one séance. To take the knee as an instance, in order to show the method of procedure in articular osteo-synovitis. Each region of the synovial membrane must be separately considered. He inserts the needle in the superior cul-de-sac in such a manner as to reach the femur on a level with the reflexion of the fungating synovial membrane, and he thus deposits the fluid on the femur itself; he endeavours always to deposit it under the periosteum. In this manner he makes four or five punctures daily, deeply in a semi-circle formed by the superior cul-de-sac; and about double this number in an adult. The principal rules to be followed in carrying out this method are: (1) Avoid injections into the cavity of the articulation. (2) That the injections should be made as far as possible at the points whence arrive the blood vessels; following up the granulations along the large ligaments, avoiding the vessels themselves wherever this can be done. (3) Avoid injections immediately beneath the skin. (4) Beginning with solutions of 1 in 40, the strength may be gradually increased to 1 in 10. In the lung he uses 1 in 40, and in the epididymis 1 in 20. (5) In tuberculosis of the ribs, iliac bones and glands, he uses 1 in 10 solution. (6) It is preferable to inject small quantities at a time, a couple of drops for example, multiplying the points of injection. (7) Before having recourse to the method, the limb should be placed in a good position and maintained there. (8) If, after a time, there are signs of recurrence, the treatment should be forthwith recommenced.

Speaking of results, M. Lannelongue confines himself to those cases in which there has been a period of three months. Twenty-two patients, were submitted to treatment and reports made. Of these, eight were cases of tubercular osteo arthritis of the knee; five arthritis of the tarsal bones; one arthritis of the elbow; two fungating patches of the chest wall with probable disease of the ribs; one patient had spina ventosa; three had multiple tubercular glands of the neck; two suffered from phthisis pulmonalis. The two latter cases must be left out of consideration, as sufficient time had not elapsed since the injections on the 23rd June to prove anything. In the case of the twenty other subjects the evolution of the processes provoked artificially, showed a marked tendency to repair.

These cases were grouped into three classes. 1st. The non-suppurating and closed tubercular lesions. In two cases, the lesions in the thorax had long since terminated; a tubercular elbow recovered all its movements; a case of disease of the tibio-tarsal joint recovered so far that he was allowed to resume walking; in two patients with tubercular glands the swelling had diminished about three-fourths. 2nd. Suppurating tubercular lesions, but closed. Two cases of white swelling in the tibio-tarsal joints had improved very rapidly. The two spina ventosa patients were cured. Some caseating glands were cured. 3rd. Suppurating and open tubercular lesions, a group comprehending two tubercular osteo-arthritis of the tibio-tarsal joints, both very advanced cases, one at any rate being a case for amputation. Both these improved greatly by the time of the report.

Such are the results obtained. M. Lannelongue pointed out that they were the effects of treatment only extending over a short time, and that it was premature to claim them as definite cures. Further observations and reports are required before any definite conclusions can be drawn, but while we think that the injections of chloride of zinc solutions, combined with surgical operations in some of the graver cases may be regarded with favour, we cannot but anticipate the conclusion that this method applied to the lung will meet with no greater success than has hitherto been obtained by intra-pulmonary injections of less irritating yet equally germicidal fluids, and that is not saying much.

